# Latest clinical recommendations on valproate use for migraine prophylaxis in women of childbearing age: overview from European Medicines Agency and European Headache Federation

**DOI:** 10.1186/s10194-018-0898-3

**Published:** 2018-08-14

**Authors:** Efstratia Vatzaki, Sabine Straus, Jean-Michel Dogne, Juan Garcia Burgos, Thomas Girard, Paolo Martelletti

**Affiliations:** 1 European Medicines Agency, 30 Churchill Place, London, E14 5EU UK; 2 Medicines Evaluation Board, Utrecht, The Netherlands; 3 PRAC member, European Medicines Agency, London, UK; 4 Department of Pharmacy, Namur Thrombosis and Haemostasis Centre - Narilis University of Namur, Namur, Belgium; 5 European Headache Federation, , https://www.ehf-org.org

**Keywords:** Migraine prophylaxis, Valproate, Pregnancy, Teratogenic risk, Foetotoxic effect, Neurodevelopmental retard, Pregnancy prevention programme, Clinical recommendations, Education, Public awareness

## Abstract

Migraine is a common and burdensome neurological condition which affects mainly female patients during their childbearing years. Valproate has been widely used for the prophylaxis of migraine attacks and is also included in the main European Guidelines. Previous (2014) European recommendations on limiting the use of valproate in women of childbearing age did not achieve their objective in terms of limiting the use of valproate in women of childbearing age and raising awareness regarding the hazardous effect of valproate to children exposed in utero. The teratogenic and foetotoxic effects of valproate are well documented, and more recent studies show that there is an even greater neurodevelopmental risk to children exposed to valproate in the womb. The latest 2018 European review from the European Medicines Agency, with the active participation of the European Headache Federation, concluded that not enough has been done to mitigate the risks associated with in utero exposure to valproate. The review called for more extensive restrictions to the conditions for prescribing, better public awareness, and a more effective education campaign in migrainous women.

## Introduction

### Migraine, gender and evolution to chronicity

Migraine is one of the most common non-communicable diseases, ranked as the most debilitating disease worldwide in under 50s by the Global Burden of Diseases 2016 [1, 2]. Migraine affects over 14% of adults worldwide, presents a higher prevalence in women (M:F ratio 1:2–3) with an estimated progression to the chronic form in about 1–4% of the population [3, 4]. In women aged 15–49 years, migraine is the top cause of years lived with disability [1, 2].

Typical migraine crises, which may be up to 3 days long, are characterised by nausea, vomiting, photophobia and phonophobia, and worsen with physical activity. Evolution into the chronic form of migraine is present mainly in female patients and its sequelae may include medication overuse [1].

Treatment of headaches in pregnancy and effects of headache medications on the child during pregnancy and breastfeeding have been reviewed recently [5]. During pregnancy and breastfeeding the preferred therapeutic strategy should be a non-pharmacological one [6].

### Valproate and migraine

Valproate has been indicated to treat epilepsy since 1967 and to treat bipolar disorders in Europe since 1995. In some European countries valproate is also indicated in prophylaxis of migraine attacks.

Guidelines regarding treatments for acute migraine and preventive therapies have been developed by the Headache and Neurology Societies [7, 8]. Valproate is included in the existing clinical guidelines of the European Federation of Neurological Societies [9] and of the European Headache Federation [10] as one of the therapies for migraine prophylaxis.

Valproate is frequently used for the treatment of pre-chronic and chronic migraine, complicated or not by medication overuse. (The prevalence of medication overuse migraine is about 2% of the entire population with chronic migraine [11] and data summarised from various studies suggest that a year after withdrawal and detoxification, 40–60% will relapse [12]).

This publication consists of four sections. The first presents the 2014 European review on the effects of valproate in children exposed in utero; the second includes the main data that question the effectiveness of the measures and recommendations agreed in 2014; the third presents the 2018 updated measures and recommendations; and finally section four summarises the main material which the physicians will use during implementation of the updated recommendations.

### European review in 2014

In October 2014, the Pharmacovigilance and Risk Assessment Committee (PRAC), a scientific committee of the European Medicines Agency (EMA) concluded a review [13] on the safety and efficacy of valproate and related substances in female children, women of childbearing potential and pregnant women. The review, which looked at all available data from non-clinical, clinical and pharmacoepidemiological studies, published literature, spontaneous reports as well as the views of relevant experts (i.e. in neurology, psychiatry, child neuropsychiatry, obstetrics etc.), led to the outcomes summarised below.

Firstly, data derived from a meta-analysis [14] (including registries and cohort studies) confirmed the already known risk of congenital malformations. Almost 11% (10.73% [95% CI: 8.16–13.29]) of children of epileptic women exposed to valproate monotherapy during pregnancy suffer from congenital malformations. The risk of major malformations in the general population is about 2–3%. The risk is dose-dependent, but a threshold dose, below which no risk exists, cannot be established based on the current data. The incidence of risk appears to be higher with valproate alone or in combination than with other anti-epileptic drugs (AEDs) alone. The most common types of malformations included neural tube defects, facial dysmorphism, cleft lip and palate, craniostenosis, cardiac, renal and urogenital defects, limb defects (including bilateral aplasia of the radius), and multiple anomalies involving various body systems.

Secondly, data have also shown that exposure to valproate in utero can have adverse effects on mental and physical development of exposed children. This risk also seems to be dose-dependent, and a threshold dose, below which no risk exists, can again not be established based on available data. The exact gestational period at risk for these effects is uncertain and the possibility of a risk throughout the entire pregnancy cannot be excluded. Studies in preschool children exposed in utero to valproate show that up to 30–40% of them experience delays in their early development such as talking and walking later, lower intellectual abilities, poor language skills (speaking and understanding) and memory problems [15–19]. Intelligence quotient (IQ) measured in school aged children (age 6) with a history of valproate exposure in utero was on average 7–10 points lower than those of children exposed to other anti-epileptics. Although the role of confounding factors cannot be excluded completely, this risk in children exposed to valproate may be independent from maternal IQ [18].

Available data show that children exposed to valproate in utero are at increased risk of autistic spectrum disorder (approximately three-fold) and childhood autism (approximately five-fold) compared with the general population. Limited data suggests that children exposed to valproate in utero may in addition be more likely to develop symptoms of attention deficit/hyperactivity disorder (ADHD) [19].

As a result, based on all the evidence available, in 2014 PRAC recommended restrictions on the use of valproate due to the risk of malformations and neurodevelopmental problems in children exposed to valproate in the womb [13].

Regarding the management of migraine prophylaxis, it was noted that there are only limited data on the efficacy of valproate. The PRAC concluded that valproate should be contraindicated in the prophylaxis of migraine attacks in pregnancy or in women of childbearing age who are not using effective methods of contraception.

Changes to the product information including contraindications, warnings and precautions, and updated information on the risks related to exposure during pregnancy to inform the clinicians and women, were agreed. Educational materials for healthcare professionals and patients were recommended.

Finally, the pharmaceutical companies for valproate were requested to perform studies to assess the effectiveness of the recommended risk minimisation measures and to further characterise the prescribing patterns for valproate with a pre- and post-implementation analysis and assessment.

### Recent data on effectiveness of the current risk minimisation measures in women of childbearing age

Fast forward a few years from the 2014 recommendations and new data started coming to light on the effectiveness of the implemented risk minimisation measures from various sources, including a drug utilisation study (DUS) and a healthcare professional survey, both performed by the pharmaceutical companies who market valproate in Europe. It is important to note that the period after implementation of the risk minimisation measures (2015–2016) is rather short and the DUS study is not completed yet. The preliminary data nonetheless suggested that prescribing behaviour changed after implementation of the risk minimisation measures. However there was additional data forthcoming from the joint healthcare professional survey (completed) which indicated that there was room for improvement regarding the knowledge and behaviour of the prescribers.

In addition, a number of national initiatives were undertaken in European countries and input from professional and patient representative organisations provided information useful for the evaluation of the effectiveness of the risk minimisation measures.

The *Agence nationale de sécurité du médicament et des produits de santé* (ANSM) in France performed a national pharmacoepidemiological study (CNAMTS study - part I) [20] covering all indications of valproate products (based on data from French national medico-administrative databases), together with a national survey conducted in pharmacies.

The results of the French study provided evidence that, despite the measures recommended following the European review in 2014, exposure of women of childbearing age to valproate had persisted in the country in the reported period including a high level of exposure during pregnancy (2 in 1000 pregnancies were exposed to valproate between 2007 and 2014).

Furthermore the results from the French survey conducted between April and June 2016 in a sample of 222 community pharmacies showed that prescribing conditions were not adhered to, especially in the bipolar disorder indication. Conditions regarding supply and use were adhered to in only 36% of the products dispensed among girls and women of childbearing age who had been prescribed valproate by a psychiatrist.

Although limited, the results from studies and surveys consistently indicated that the risk minimisation measures of 2014 did not have a satisfactory impact on prescribing patterns.

In addition the results also indicated that the switching rates for transfer to other medication in patients at risk were not high enough.

Although these observations were identified mainly in the epilepsy and bipolar disorder indications, there are no reasons or data to indicate that these results cannot be extrapolated also to the indication of migraine prophylaxis.

Data from all these different sources further confirm the need for improved risk communication among prescribers and patients.

### Reinforcement of the risk minimisation measures and update of the clinical recommendations following the 2018 review

In the light of the above the PRAC carried out a further review of the risks and measures to manage them, resulting in new recommendations issued in 2018 [21].

Here only those recommendations relevant to the indication on prophylaxis of migraine are discussed, and specifically the measures that require the involvement of the physician for their implementation in collaboration with the patient. Further details of the full recommendations can be found in the publicly available assessment report of the latest 2018 European review [21].

Updates to the product information were deemed to be necessary in order to reduce valproate exposure during pregnancy. The risks of congenital malformations and neurodevelopmental are, and will always remain inherent to valproate, and cannot be reduced or eliminated. However by reducing or minimizing exposure during pregnancy these risks can be mitigated.

The PRAC recommended the contraindication of valproate treatment during pregnancy in the indication of bipolar disorders [21, 22] and maintained its 2014 recommendation of contraindication of valproate treatment during pregnancy in the prophylaxis of migraine attacks [13], in order to protect the unborn child from a major risk of congenital malformations and neurodevelopmental disorders.

The PRAC also recommended several measures as part of pregnancy prevention programme (PPP) to adhere to.

First of all, the individual patient circumstances should be evaluated in each case to guarantee engagement. The treating physician should discuss the therapeutic options with each patient and ensure her understanding of the risks of treatment with valproate and the measures needed to minimise these risks. The patient needs to acknowledge that she has understood the hazards and necessary precautions associated with valproate use and sign a risk acknowledgement form.

During the consultation the patient must understand the need to undergo pregnancy testing prior to initiation of treatment and during treatment, as needed.

It is essential to ensure that the patient is counselled regarding contraception. Assessment also is needed that the patient is capable of complying with the requirement to use effective contraception without interruption during the entire duration of treatment with valproate. Before contraception is discontinued, the patient must consult her physician as soon as she is planning pregnancy to ensure timely discussion of switching to alternative treatment options prior to conception and so the need for migraine treatment can be re-evaluated. In case of unplanned pregnancy the patient should urgently consult with her physician to organise the discontinuation of the migraine treatment.

As the personal circumstances of the patient may change, regular (at least annual) reviews of treatment need to be performed by a specialist experienced in the management of migraine.

These PPP conditions also concern women who are not currently sexually active unless the prescriber considers that there are compelling reasons to indicate that there is no risk of pregnancy.

Educational measures are necessary in order to ensure that both the clinical community and patients are informed about the risks of valproate in pregnant women and women of childbearing age, and about the measures necessary to eliminate the risk of exposure on valproate in pregnancy.

### Educational materials

In this publication, only the material relevant to prescribers is mentioned. A full list of the educational materials can be found in the published assessment report [21].

#### Guide for healthcare professionals

The development of an improved healthcare professional guide is due following the 2018 review. This guide will explain the PPP and its conditions. It will explain the requirements prior to starting treatment with valproate, the modalities for annual re-assessment of the need for valproate therapy, and discontinuation or switching to alternative treatment options for female children who experience menarche and women of childbearing potential.

Recommendations on switching or discontinuing valproate treatment, as well as recommendations on pregnancy planning, will support provision of this information to patients.

Actions to mitigate the risks associated with the use of valproate in case of pregnancy will also be included.

This guide should allow prescribers to familiarise themselves with the more recent data on disorders of development in the exposed child, provide information about the risks of valproate monotherapy and poly-therapy and a description of the roles of different healthcare professionals. It should also provide instructions to the prescribers on the distribution of the patient guide and the completion of the risk acknowledgment form.

#### Annual risk acknowledgment form

As part of the PPP, an annual risk acknowledgment form will be made available to support the transmission of information to the patient and foster the dialogue between the prescriber and the patient. This form will ensure that the information has been given and that it is understood by the patient.

The form will include a checklist for prescribers and patients (or carers). The checklist is intended to be used by physicians at the time of treatment initiation, and at the annual review to facilitate discussion with female patients about the suitability of valproate treatment and its risks.

This acknowledgment form will cover information about the risk to the unborn baby in case of in utero exposure to valproate, the need for negative pregnancy test at treatment initiation, the need for effective contraception without interruption throughout the entire duration of treatment with valproate (if of childbearing potential). It will also require that the patient contacts her physician once she decides to plan for a pregnancy, to ensure timely discussion of alternative treatment options prior to conception and before contraception is discontinued, and that she contacts her doctor immediately for an urgent treatment review in case of suspected or inadvertent pregnancy.

#### Guide for patients

The development of an improved guide for women who are being prescribed valproate and are able to get pregnant, is also in progress. Although this document is addressed to patients it is summarised here as treating physicians may be requested to provide this guide directly to their patient.

The guide will provide comprehensive information on risks to the unborn child due to in utero exposure to valproate, the details of the PPP and the required actions in case of pregnancy or intention to become pregnant. In order to provide adequate information, it will address different situations in the patient journey and be age-appropriate: from first prescription, women continuing valproate treatment but not trying to have a child, women of childbearing potential continuing valproate treatment and considering trying to have a child, and pregnant women (unplanned pregnancy). Information about the annual risk acknowledgement form and the patient card which has also been developed should be included in the patient guide.

## Conclusions

Migraine and chronic headaches occur frequently in the population and more specifically in women. In case of pregnancy the treatment of migraine may be complicated due to the risk of certain medications. For the preventive treatments there are fewer options available to physicians.

Valproate has a place in the pharmaceutical armamentarium for the prophylaxis of migraine attacks, according to several clinical guidelines.

Valproate treatment should be initiated with caution in young females and women of child-bearing potential in view of the risks to the foetus in the event of exposure to valproate during pregnancy.

In view of the risks for the unborn child when exposed to valproate in utero, valproate should only be used as last line medication for prophylaxis of migraine attacks in female children, adolescents and women of childbearing potential.

Adherence to the PPP is essential. Pregnancy should be excluded before start of any treatment with valproate, and as appropriate later on. The patient should be counselled regarding contraception, but also assessed to ensure she can comply with the use of effective contraception without interruption during the entire duration of treatment with valproate. In case of unplanned pregnancy, valproate treatment should be discontinued as soon as possible. The information on the risks in case of an unplanned pregnancy should be explained to the patient at the first prescription and then at least annually for the duration of the treatment.

Together, the clinician and patient should discuss the need for regular (at least annual) review of her treatment by a specialist experienced in the management of migraine. This discussion should be documented via the risk acknowledgement form.

The importance of the patient consulting her physician as soon as she is planning a pregnancy needs to be emphasised. This will enable a timely discussion to take place between the physician and the patient to ensure switching to alternative treatment options prior to conception and before contraception is discontinued.

Clinical guidelines need to be reviewed and, potentially, updated to further improve the awareness of physicians on the risks of valproate.

Finally, continuous interaction between the European Medicine Agency and European Headache Federation, as an eligible healthcare professional organisation dedicated to migraine, can facilitate the reduction of unnecessary exposure to valproate in migrainous women of childbearing age, via provision of better education materials for both physicians and patients suffering from migraine.

## Abbreviations

ADHD: Attention deficit hypersensitivity Disorder
AED: Anti-epileptic drugs
ANSM: Agence Nationale de la Sécurité du Médicament et des produits de santé
CNAMTS: Caisse nationale de l’assurance maladie des travailleurs salariés
DUS: Drug utilisation study
EMA: European Medicines Agency
IQ: Intelligence quotient
PPP: Pregnancy prevention programme
PRAC: Pharmacovigilance and Risk Assessment Committee
